# Stability of Hold and Postural Control During Static Hold Assessment Can Provide Valuable Information for Biathlon Standing Shooting but Aiming Strategy Should Be Acknowledged

**DOI:** 10.1002/ejsc.70045

**Published:** 2025-08-22

**Authors:** Miika Köykkä, Marko S. Laaksonen, Keijo Ruotsalainen, Vesa Linnamo

**Affiliations:** ^1^ Faculty of Sport and Health Sciences University of Jyväskylä Jyväskylä Finland; ^2^ Finnish Institute of High Performance Sport KIHU Jyväskylä Finland; ^3^ Department of Health Sciences Swedish Winter Sports Research Centre Mid Sweden University Östersund Sweden

**Keywords:** biomechanics, optoelectronics, performance, rifle shooting, technique

## Abstract

This study investigated stability of hold and postural control in a static holding task and a biathlon standing shooting task, and shooting performance, examining the associations between these tasks and potential differences between biathletes employing a hold‐based (HBS) or timing‐based (TBS) aiming strategy. Twenty‐two biathletes completed a static holding test in the standing shooting posture (Holding) and a biathlon standing shooting test (Shooting) using dry shots. Postural control was evaluated using force platforms, and stability of hold was assessed using a Vicon motion capture system (Holding) and a Noptel training device (Shooting). Of the stability of hold measures, links between the tasks were observed in aiming point vertical standard deviation (*R* = 0.78) and vertical mean velocity (*R* = 0.74) in HBS and in vertical mean velocity (*R* = 0.62) in TBS (all *P* < 0.05). Regardless of aiming strategy, most postural control measures in Holding correlated with their counterparts in Shooting (*R* = 0.48 to 0.94 and *P* < 0.05) and shooting performance (hit point distance from the centre of the target) correlated with stability of hold measured during Holding (*R* = 0.74 and *P* < 0.001). Among the postural control measures, shooting performance was only clearly linked to front leg postural control measured during Holding in TBS (*R* = 0.87 and *P* < 0.003). These findings suggest that static holding ability and postural control are important in biathlon standing shooting regardless of aiming strategy but aiming dynamics during Holding align more closely with the practices of HBS during their shooting.

## Highlights

- Although static stability of hold is important for shooting performance regardless of aiming strategy, it is only clearly linked to stability of hold during shooting for those using a hold‐based aiming strategy and those using a timing‐based strategy may require a more strategy‐specific test.
- Postural control magnitudes measured during Holding provide a good indication of those in Shooting.
- The static holding protocol provides an efficient and valid way to assess key characteristics such as isolated stability of hold and postural control.

## 1 Introduction

Biathlon combines cross‐country skiing and rifle shooting, with success depending on skiing speed, shooting accuracy and time spent on the shooting range (Björklund et al. 2022; Björklund and Laaksonen 2022; Luchsinger et al. 2018, 2019, 2020). Unlike rifle precision shooters, biathletes shoot although physically exhausted, aiming to hit five targets (diameter 4.5 cm in the prone and 11.5 cm in the standing posture) quickly during each shooting bout.

In the standing shooting posture, biathletes stand on both legs holding the rifle with both hands. Research shows that stability of hold (Ihalainen et al. 2018; Sattlecker et al. 2014, 2017) and cleanness of triggering (Ihalainen et al. 2018) are associated with shooting performance. Postural control is a crucial factor influencing shooting performance both directly and indirectly through other components (Ihalainen et al. 2018; Sattlecker et al. 2014, 2017). Furthermore, rifle sway (Sattlecker et al. 2014) and postural control magnitudes (Ihalainen et al. 2018) during standing shooting seem to discriminate between different levels of biathletes.

Previous studies have explored the associations between stability of hold, postural control and shooting performance (Ihalainen et al. 2018; Sattlecker et al. 2014, 2017) during shooting. However, these studies typically analysed data collected 0.5–0.6 s prior to triggering, potentially confounding the results due to other influencing factors such as triggering technique.

In practice, biathletes may engage in up to 130 dry shooting sessions annually (Laaksonen et al. 2018), often focusing on isolated hold training in the shooting posture. This hold training typically consists of several prolonged periods of static hold without the intention to trigger. Despite the widespread use of such training, there remains a gap in understanding the impact of stability of hold and postural control during static hold on actual shooting.

Exploring the association between static hold and shooting could clarify whether stability during static hold is an essential skill for biathletes. Additionally, this could demonstrate the effectiveness of a simplified static hold test to assess stability and postural control in isolation free from confounding variables. Implementing such a test could enable coaches and athletes to efficiently track progress and rapidly evaluate adjustments in posture or rifle settings without the need for extensive full‐scale shooting assessments previously described (Ihalainen et al. 2018; Sattlecker et al. 2014, 2017).

Strong correlations have been identified between postural control magnitudes and rifle movements during a static holding task (Sadowska et al. 2019), suggesting that greater postural control magnitudes are associated with increased rifle movement. During quiet standing, biathletes exhibit lower postural control magnitudes compared to nonathletes (Sadowska et al. 2020), indicating their superior postural balance in a nonsport‐specific task. However, biathlon shooting performance and aiming point movements were not recorded in either study. Studies on biathlon standing shooting have indicated no differences in rifle accelerations between the most and least accurate shots (Köykkä et al. 2023) or between shooting sets after skiing at varying intensities (Žák et al. 2019). These findings imply that biathletes could compensate for rifle movements caused by postural sway by rotating the rifle horizontally and/or vertically to maintain steady aim. Thus, even though the rifle moves in space alongside postural sway, the aiming point may remain stable on the target. Therefore, a comprehensive understanding of the link between static holding and biathlon shooting requires examining both shooting performance and aiming point movements, which have not been addressed in the previous studies (Sadowska et al. 2019; Sadowska et al. 2020).

It is also important to consider aiming strategies while analysing the associations between stability of hold, postural sway and shooting performance during static holding and biathlon shooting tasks, particularly in comparisons across tasks. When interpreting biathlon shooting analyses using the aiming strategy framework, as thoroughly described by Köykkä (2024), biathletes are categorised based on how they approach the target during aiming. In a hold‐based strategy, the biathlete attempts to stabilise the aiming point within the hit area well before triggering, whereas a timing‐based strategy involves pulling the trigger while the aim is still moving towards the centre (Köykkä 2024). This differs from the behaviour of rifle precision shooters, who stabilise the aiming point close to the target centre prior to triggering and hold stability distinguishes between skill levels (Goodman et al. 2009; Zatsiorsky and Aktov 1990). Thus, they show similar aiming strategies. Köykkä et al. (2021) suggest that, in biathlon standing shooting, the metrics that best describe shooting technical level depend on the chosen strategy. During shooting, those using a hold‐based approach benefit from having a brief period of stable hold within the hit area and the ability to consistently aim at the centre during that hold similar to the rifle precision shooters' strategy. Conversely, biathletes adopting a timing‐based approach may perform better with slower movement of the aiming point towards triggering and precise timing of the trigger pull while still moving towards the centre. However, lower postural control magnitudes are associated with better shooting performance, regardless of the strategy (Köykkä et al. 2021).

The primary purpose of this study was to investigate biathlon standing shooting performance, focusing on stability of hold and postural control during a static holding task and a biathlon standing shooting task. The aim was to understand the associations between these tasks and the impact of aiming strategy. The first research question addressed whether aiming strategy affects stability of hold and postural control magnitudes during both tasks. We hypothesised that biathletes using a hold strategy would have better stability of hold during shooting compared to those using a timing strategy, whereas both groups would show similar stability of hold during static holding. Additionally, we hypothesised postural control magnitudes to be similar across both strategies. The second research question explored whether biathlon standing shooting performance is associated with stability of hold and postural control measured during the static holding task. We hypothesised that better stability of hold and lower postural control magnitudes during holding would correlate with better shooting performance. The third research question examined whether stability of hold and postural control magnitudes during the holding task are linked to their counterparts during the shooting task. We hypothesised that these measures would be linked in biathletes using a hold‐based strategy. In those employing a timing‐based strategy, we hypothesised that stability of hold measures are not but postural control magnitudes are linked across tasks.

## 2 Methods

### 2.1 Participants

Eligibility required previous participation in major international competitions and current membership in an official national team within their age group. This ensured that all participants were among the top performers in their age group within their nation and classified as tier 3 to 4 athletes (McKay et al. 2022). From the 27 invited biathletes, 22 (8 females) volunteered for this study. At the time of the testing, participants competed in Youth (under 19 years; *n* = 7), Junior (under 22 years; *n* = 10) or Men/Women (*n* = 5) categories (International Biathlon Union 2023). All had prior experience with the testing protocols.

The study followed the Declaration of Helsinki guidelines and was approved by the University of Jyväskylä Ethics Committee (6 June 2019). Participants were informed about study's purpose, nature and potential risks and they gave written consent before participating.

### 2.2 Experimental Overview

#### 2.2.1 Overall Design

This study utilised a cross‐sectional observational design. During a single visit to a biathlon shooting laboratory, participants performed static holding and dry biathlon shooting tasks in the standing posture using their personal biathlon rifles. They were instructed to avoid strenuous physical activity before the tests.

#### 2.2.2 Static Holding Task (Holding)

Each participant completed four 45‐s sets of static holding using the standing shooting posture, with 30‐s breaks between sets. While maintaining the posture and aiming at the target, stability of hold and postural sway were measured for 10 s at the 10‐ and 35‐s marks, following a verbal cue. Upon the start cue, participants mimicked the start of shooting, maintained their aim at the centre of the target and held their breath until the stop cue.

#### 2.2.3 Biathlon Standing Shooting Task (Shooting)

Before the task, rifles were zeroed. Participants then completed 10 single shots and two to four 5‐shot sets, familiarising themselves with the shooting spot. The shooting task involved six 5‐shot sets, with approximately 30‐s breaks in between, while in a resting state and adopting their usual competition shooting rhythm and technique. In each set, the participant initially stood behind the shooting mat, transitioned into the shooting posture, executed five dry shots without ammunition into a scaled target at 10 m distance and returned to the initial standing posture. Shooting performance, stability of hold and postural control were measured for each shot.

### 2.3 Data Collection

Postural control was evaluated using ground reaction force and moment time series data from two AMTI BP4181068RS‐1000 force platforms (Advanced Mechanical Technology Inc., Watertown, USA). These platforms, connected to a GEN‐5 amplifier, were positioned under each leg and sampled data at 400 Hz. The centre of pressure (COP) coordinates were computed for the front (the leg closer to the target) and rear legs (the leg further from the target) and the whole body (details in Appendix 1). In the biathlon standing shooting posture, the legs are oriented so that an imaginary line connecting the feet is approximately aligned with the shooting direction. COP coordinate data were filtered using a zero‐lag fourth‐order digital low pass filter, with a 10 Hz cutoff frequency.

For Holding, the 3D positions of two reflective markers placed 220 mm apart beneath the rifle barrel were recorded at 150 Hz using a Vicon motion capture system (Vicon Motion Systems Ltd, Oxford, UK) equipped with five Vicon T40‐S cameras. These trajectories were filtered using a zero‐lag first‐order low‐pass Butterworth filter with an 8 Hz cutoff frequency. The line formed by these markers' trajectories was projected onto a plane perpendicular to the shooting line at 50 m distance. The point of intersection between this line and the plane was recognised as the aiming point in both the left–right and up–down dimensions of the plane. (Details in Appendix 2).

During Shooting, shooting performance and aiming point movement were captured at 67 Hz using a Noptel ST 2000 training device (Noptel Inc., Oulu, Finland). This device comprised an 80 g optical transmitter–receiver unit attached to the barrel of the rifle and a reflector attached around the targets.

Force platform, trigger force and external microphone data were collected and synchronised using the Coachtech online measurement and feedback system (University of Jyväskylä, Vuokatti, Finland) (Ohtonen et al. 2016). During Holding, an external trigger signal from the Coachtech software to the motion capture system (Vicon) marked the start of capturing force platform data. During Shooting, microphone data identified the triggering moment and synchronised between aiming (Noptel) and force platform data, matching the triggering moment detected by the Noptel software with the microphone pulse generated during triggering. Trigger force data from a pressure sensor (FSR 402, Interlink Electronics Inc., Irvine, CA, USA) attached to the rifle trigger, collected synchronously with the Coachtech system at 400 Hz, was utilised to filter out shots incorrectly detected by the Noptel system, such as those caused by rifle reloads (Köykkä et al. 2022). The Coachtech software for signal processing was created using the LabVIEW programing environment (National Instruments Corporation, Austin, TX, USA).

### 2.4 Variables

COP and aiming coordinates during the 10‐s holds in Holding and the last 0.6 s before each triggering in Shooting were analysed using custom‐made scripts in MATLAB R2023a (The MathWorks Inc., Natick, MA, USA) to derive variables characterising stability of hold and postural control (Table 1). For Holding, trace lengths of COP and aiming coordinate signals were computed during the 10‐s hold periods, with the shortest consecutive 5‐s traces used for analyses. For Shooting, the final 0.6‐s traces before each triggering were extracted. Shooting performance was assessed as the hit point distance (millimetres) from the target centre.

**TABLE 1:** Variables characterising stability of hold and postural control.

Variable (unit)	Description
95% CE A (mm^2^)	95% confidence ellipse area
X SD and Y SD (mm)	Standard deviation in the X and Y directions
X MV, Y MV and R MV (mm/s)	Mean velocity in the X, Y and resultant directions

Abbreviations: X, cross‐shooting direction (postural control)/left–right (stability of hold); Y, shooting direction (postural control)/up–down (stability of hold).

COP variables, representing the magnitude of postural control, were computed for the whole body as well as separately for the front leg and rear legs. The interpretation of the variables is based on previous research by Winter et al. (1993), (1996). Hip muscles primarily regulate weight distribution between limbs, whereas ankle muscles control sway (Winter et al. 1993). When weight is redistributed, the whole body COP oscillates along a line connecting the individual COPs under each foot (Winter et al. 1996). Significant deviations from a side‐by‐side foot position along the shooting direction are uncommon, based on the authors' biathlon coaching experience. Therefore, whole body COP magnitudes in the shooting direction were interpreted as oscillations in weight distribution, whereas magnitudes in the cross‐shooting direction represented whole‐body sway controlled by the ankles. Front and rear leg COP magnitudes further reflected postural control by the individual ankles in each respective direction.

Additionally, the aiming approach velocity in Shooting 0.4 to 0.2 s before triggering was computed for each participant as detailed in a previous study (Köykkä et al. 2021). Briefly, the distance from the centre of the target signal values of the shots at each time instant from 0.4 to 0.2 s before triggering were averaged for each participant. Subsequently, approach velocity was defined as the mean rate of change in the average signal.

### 2.5 Missing Data

Due to technical issues with force platforms and marker visibility on the rifle barrel, resulting in abnormally noisy signals in some tests. Therefore, trials with abnormal signals were excluded, determined via interquartile range criteria, ensuring data quality and comparability. Initially, trace length computation was applied to the aiming and COP coordinate data. Outliers were identified using the interquartile method, with a coefficient of 2.2 to define outlier limits for the trace lengths, following Hoaglin and Iglewicz (1987).

In total, COP data from 6 out of 88 sets (6.8%) and aiming data from 4 out of 88 sets (4.5%) were identified as outliers in Holding and COP data from 59 out of 660 shots (8.9%) were identified as outliers in Shooting. Participants with incomplete data were excluded on an analysis‐by‐analysis basis, resulting in varying numbers of participants per analysis. The sample size for each analysis is reported alongside the results.

### 2.6 Statistical Analysis

Statistical analysis was conducted in R version 4.4.0 (R Core Team 2024). The alpha level of 0.05 was applied for all analyses.

Test mean values for all variables of the eight measured hold periods in Holding and all shots in Shooting were computed and used in analyses. Mean ± standard deviation describe the distribution and extent of variation within the data and are reported where applicable. The Shapiro–Wilk test assessed data distribution, and the Levene's test assessed the equality of variances, utilising the ‘stats’ package version 4.4.0 (R Core Team 2024).

Participants were categorised into subgroups based on aiming approach velocity. Those below median velocity formed the hold‐based aiming strategy group (HBS), whereas the remaining participants comprised the timing‐based aiming strategy group (TBS) as defined by Köykkä et al. (2021). Differences in age, biathlon experience, shooting performance (Hit dist) and approach velocity between HBS and TBS were investigated utilising the ‘stats’ package (R Core Team 2024), after ensuring normal distribution and equal variances of residuals within each group.

A mixed‐design ANOVA, using the ‘car’ package version 3.1–2 (Fox and Weisberg 2019), examined the effect of the between‐subjects factor (Strategy: HBS and TBS), the within‐subjects factor (Task: Holding and Shooting) and their interaction on stability of hold and postural control variables. Prior to the analysis, residuals' normality and equality of variances were confirmed. Significant Strategy × Task interactions underwent post hoc pairwise comparisons using the ‘emmeans’ package version 1.9.0 (Lenth 2024). Partial eta squared (η_p_^2^) values determined the variance explained by Strategy, Task and their interaction. Cohen's *d* effect sizes assessed the magnitude of differences between conditions, focusing on the Strategy × Task interaction, and were categorised as large (|*d*| ≥ 0.80), medium (0.50 ≤ |*d*| < 0.80) or small (0.20 ≤ |*d*| < 0.50) in pairwise comparisons (Cohen 1988).

To assess associations between tasks, Pearson (normally distributed data) or Spearman (abnormally distributed data) correlation coefficients (Rp and Rs, respectively) were computed utilising the ‘stats’ package (R Core Team 2024). If ANOVA indicated significant Strategy or Strategy × Task interaction effects, HBS and TBS were analysed separately. Otherwise, the entire sample was analysed together. A complementary correlation analysis with separated groups was performed between shooting performance and front leg COP 95% CE A during Holding, as the plot indicated potential strategy‐associated trends. Correlation coefficients were interpreted as very weak (|*R*| < 0.2), weak (0.2 ≤ |*R*| < 0.4), moderate (0.4 ≤ |*R*| < 0.6), strong (0.6 ≤ |*R*| < 0.8) or very strong (|R| ≥ 0.8) (Evans 1996).

*p* values were adjusted for multiple comparisons using the false discovery rate method (Benjamini and Hochberg 1995). To estimate the robustness of effect size estimations, bootstrap resampling with 1000 iterations was performed using the ‘boot’ package version 1.3.28.1 (Davison and Hinkley 1997). This method derived 95% confidence intervals (CIs) for effect size measures, which are reported as η^2^ [95% CI], *d* [95% CI], Rp [95% CI] and Rs [95% CI].

## 3 Results

No differences were observed between the groups for age (HBS 19 ± 2 years vs. TBS 21 ± 2 years; t(20) = 1.82, *p* = 0.17 and *d* = 0.78 [−0.03, 1.97]), biathlon experience (8 ± 2 years vs. 9 ± 3 years; t(20) = 1.20, *p* = 0.33 and *d* = 0.51 [−0.29, 1.30]) or shooting performance (Hit dist 34 ± 7 mm vs. 31 ± 8 mm; t(20) = −0.95, *p* = 0.35 and *d* = −0.41 [−1.63, 0.28]). HBS demonstrated lower approach velocity compared to TBS (57 ± 14 vs. 111 ± 23 mm/s; t(20) = 6.81, *p* < 0.001 and *d* = 2.91 [2.22, 4.71]).

### 3.1 Stability of Hold

Table 2 presents descriptive and ANOVA analyses for stability of hold measures. TBS demonstrated lower resultant, horizontal and vertical mean velocities (R MV, X MV and Y MV) and horizontal standard deviation (X SD). Confidence ellipse area (95% CE A) and vertical standard deviation (Y SD) did not differ between the groups.

**TABLE 2:** Descriptive statistics of stability of hold variables for aiming (mean ± SD) in both the hold and timing groups during the holding and shooting tasks, and the two‐way mixed ANOVA (Strategy, Task, and Strategy × Task) and post hoc pairwise comparisons summary statistics.

	95% CE A [cm^2^]	R MV [mm/s]	X SD [mm]	X MV [mm/s]	Y SD [mm]	Y MV [mm/s]
All (*n* = 21)						
Holding	151 ± 66	379 ± 71	34 ± 7	269 ± 49	23 ± 6	212 ± 43
Shooting	45 ± 14	251 ± 45	19 ± 3	157 ± 29	18 ± 5	161 ± 32
HBS (*n* = 10)						
Holding	175 ± 55	414 ± 67	38 ± 5	290 ± 46	25 ± 5	236 ± 42
Shooting	44 ± 12	268 ± 48	19 ± 3	171 ± 30	16 ± 2	170 ± 34
TBS (*n* = 11)						
Holding	130 ± 70	348 ± 61	31 ± 6	250 ± 45	22 ± 7	190 ± 33
Shooting	46 ± 17	235 ± 38	18 ± 3	145 ± 24	20 ± 6	154 ± 29
Main effect of Task						
F (1, 19)	136.98	138.38	204.68	149.53	24.34	71.69
*p*	**<** **0.001**	**<** **0.001**	**<** **0.001**	**<** **0.001**	**<** **0.001**	**<** **0.001**
η_p_^2^ [95% CI]	0.88 [0.81, 0.92]	0.88 [0.83, 0.91]	0.92 [0.88, 0.95]	0.89 [0.84, 0.92]	0.56 [0.39, 0.84]	0.79 [0.71, 0.87]
Main effect of Strategy						
F (1, 19)	0.51	5.48	6.58	5.99	0.10	4.94
*p*	0.484	**0.030**	**0.019**	**0.024**	0.760	**0.039**
η_p_^2^ [95% CI]	0.03 [0.00, 0.23]	0.22 [0.02, 0.65]	0.26 [0.05, 0.57]	0.24 [0.03, 0.58]	0.01 [0.00, 0.05]	0.21 [0.01, 0.57]
Interaction effect						
F (1, 19)	1.03	2.32	8.10	0.60	9.93	5.66
*p*	0.322	0.144	**0.010**	0.448	**0.005**	**0.028**
η_p_^2^ [95% CI]	0.05 [0.00, 0.28]	0.11 [0.00, 0.39]	0.30 [0.05, 0.62]	0.03 [0.12, 0.58]	0.34 [0.12, 0.58]	0.23 [0.02, 0.46]

*Note:* Bold *p* values indicate *p* < 0.05.

Abbreviations: η_p_^2^, partial eta squared; CE A, confidence ellipse area; HBS, hold‐based aiming strategy group; MV, mean velocity; R, resultant; SD, standard deviation; TBS, timing‐based aiming strategy group; X, horizontal (left–right); Y, vertical (up–down).

Strategy × Task interaction effects were observed in X SD, Y SD and Y MV. Pairwise comparisons revealed that both X SD and Y MV were larger during Holding compared to Shooting in both groups, with a more pronounced difference between the tasks in HBS (X SD: *p* < 0.001 and *d* = 8.38 [7.02, 14.21]; Y MV: *p* < 0.001 and *d* = 5.30 [5.01, 6.59]) than in TBS (X SD: *p* < 0.001 and *d* = 5.87 [5.56, 7.51]; Y MV: *p* = 0.003 and *d* = 3.12 [2.56, 8.11]). Additionally, TBS demonstrated lower X SD than HBS during Holding (*p* < 0.048 and *d* = 2.06 [1.22, 4.29]), with no difference observed during Shooting (*p* = 0.86 and *d* = 0.55 [−0.20, 1.67]). Furthermore, Y SD was higher in Holding compared to Shooting in HBS (*p* < 0.001 and *d* = 3.95 [2.80, 9.06]). No difference was observed between the tasks in TBS (*p* = 0.58 and *d* = 0.91 [−0.09, 2.83]) (Table 2).

**FIGURE 1:**
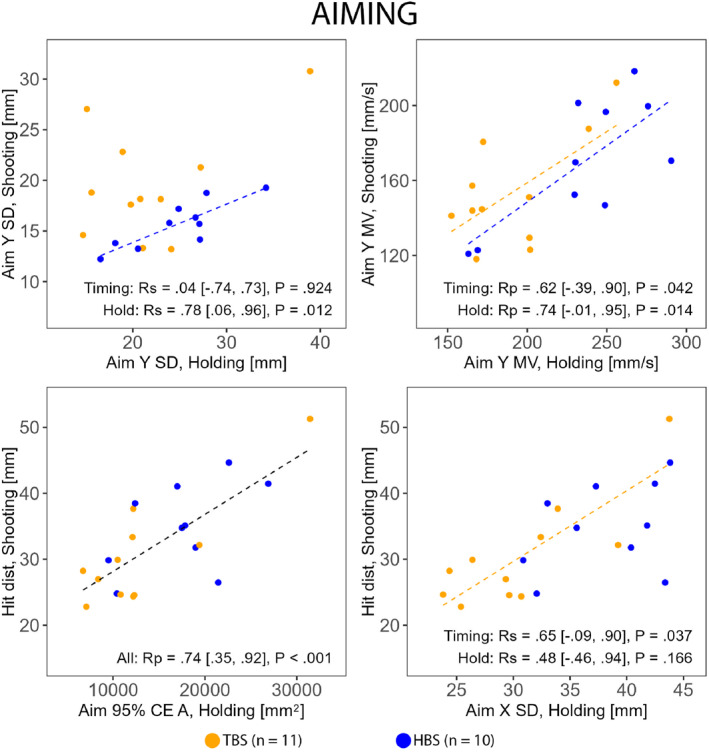
Correlations (Rp Pearson and Rs Spearman) between selected corresponding stability of hold measures during Holding and Shooting and between stability of hold measures during Holding and shooting performance (Hit dist) in HBS (hold‐based aiming strategy group) and TBS (timing‐based aiming strategy group). CE A, confidence ellipse area; MV, mean velocity; SD, standard deviation; X, horizontal (left–right); Y, vertical (up–down).

Stability of hold measures in Holding were associated with their counterparts in Shooting and with shooting performance (Figure 1). Notably, only HBS exhibited associations between the tasks in the vertical standard deviation of the aiming point, whereas only TBS exhibited an association between the horizontal standard deviation of the aiming point and shooting performance (Figure 1). For all correlations, see Supporting Information S1: Supplemental Material.

### 3.2 Postural Control

Tables 3, 4, 5 present the ANOVA analyses for whole body, front leg, and rear leg postural control magnitudes, respectively. TBS demonstrated lower whole body COP R MV and X MV. No strategy effects were observed for front or rear leg postural control magnitudes.

**TABLE 3:** Descriptive statistics of postural control variables for the whole body (mean ± SD) in both the hold and timing groups during the holding and shooting tasks, and the two‐way mixed ANOVA (Strategy, Task and Strategy × Task) and post hoc pairwise comparisons summary statistics.

	95% CE A [mm^2^]	R MV [mm/s]	X SD [mm]	X MV [mm/s]	Y SD [mm]	Y MV [mm/s]
All (*n* = 20)						
Holding	28.96 ± 8.72	11.30 ± 2.47	0.72 ± 0.12	7.79 ± 2.18	2.18 ± 0.40	6.35 ± 1.44
Shooting	5.89 ± 1.80	12.64 ± 2.06	0.55 ± 0.10	7.38 ± 1.95	0.70 ± 0.09	5.91 ± 0.91
HBS (*n* = 11)						
Holding	31.93 ± 8.75	12.33 ± 2.65	0.77 ± 0.11	8.90 ± 2.25	2.24 ± 0.34	6.52 ± 1.53
Shooting	6.14 ± 1.77	13.44 ± 2.08	0.57 ± 0.12	8.21 ± 2.12	0.68 ± 0.07	5.95 ± 0.86
TBS (*n* = 9)						
Holding	25.33 ± 7.62	10.05 ± 1.58	0.66 ± 0.10	6.43 ± 1.11	2.11 ± 0.47	6.14 ± 1.39
Shooting	5.57 ± 1.90	11.66 ± 1.66	0.52 ± 0.07	6.36 ± 1.15	0.73 ± 0.11	5.86 ± 1.01
Main effect of task						
F (1, 18)	503.50	13.62	63.51	3.13	631.38	1.23
*p*	**<** **0.001**	**0.002**	**<** **0.001**	0.094	**<** **0.001**	0.282
η_p_^2^ [95% CI]	0.97 [0.95, 0.98]	0.43 [0.20, 0.80]	0.78 [0.66, 0.87]	0.15 [0.01, 0.36]	0.97 [0.96, 0.99]	0.06 [0.00, 0.33]
Main effect of strategy						
F (1, 18)	2.59	5.62	3.96	7.74	0.01	0.35
*p*	0.125	**0.029**	0.621	**0.012**	0.934	0.564
η_p_^2^ [95% CI]	0.13 [0.00, 0.34]	0.24 [0.03, 0.48]	0.18 [0.01, 0.37]	0.30 [0.08, 0.56]	0.00 [0.00, 0.00]	0.02 [0.00, 0.19]
Interaction effect						
F (1, 18)	2.65	0.47	1.57	1.99	2.32	0.17
*p*	0.121	0.503	0.227	0.175	0.145	0.685
η_p_^2^ [95% CI]	0.13 [0.00, 0.43]	0.03 [0.00, 0.15]	0.08 [0.00, 0.30]	0.10 [0.00, 0.32]	0.11 [0.00, 0.42]	0.01 [0.00, 0.09]

*Note:* Bold *p* values indicate *p* < 0.05.

Abbreviations: η_p_^2^, partial eta squared; CE A, confidence ellipse area; HBS, hold‐based aiming strategy group; MV, mean velocity; R, resultant; SD, standard deviation; TBS, timing‐based aiming strategy group; *X*, cross‐shooting direction; Y, shooting direction.

**TABLE 4:** Descriptive statistics of postural control variables for the front leg (mean ± SD) in both the hold and timing groups during the holding and shooting tasks, and the two‐way mixed ANOVA (Strategy, Task and Strategy × Task) summary statistics.

	95% CE A [mm^2^]	R MV [mm/s]	X SD [mm]	X MV [mm/s]	Y SD [mm]	Y MV [mm/s]
All (*n* = 20)						
Holding	4.37 ± 2.03	7.45 ± 1.64	0.68 ± 0.40	2.58 ± 0.98	2.15 ± 0.55	6.43 ± 1.50
Shooting	0.88 ± 0.33	8.24 ± 1.59	0.21 ± 0.11	2.33 ± 0.78	0.68 ± 0.14	6.18 ± 1.38
HBS (*n* = 11)						
Holding	4.56 ± 2.18	7.55 ± 1.83	0.58 ± 0.31	2.48 ± 0.90	2.22 ± 0.46	6.57 ± 1.71
Shooting	0.86 ± 0.29	8.35 ± 1.79	0.17 ± 0.06	2.13 ± 0.54	0.68 ± 0.16	6.47 ± 1.74
TBS (*n* = 9)						
Holding	4.14 ± 1.93	7.33 ± 1.48	0.79 ± 0.48	2.70 ± 1.12	2.06 ± 0.67	6.26 ± 1.27
Shooting	0.91 ± 0.38	8.10 ± 1.40	0.27 ± 0.13	2.57 ± 0.99	0.67 ± 0.10	5.83 ± 0.67
Main effect of Task						
F (1, 18)	316.75	4.34	93.91	3.75	305.07	0.50
*p*	**<** **0.001**	0.052	**<** **0.001**	0.069	**<** **0.001**	0.487
η_p_^2^ [95% CI]	0.95 [0.92, 0.96]	0.19 [0.02, 0.44]	0.84 [0.74, 0.94]	0.17 [0.01, 0.40]	0.94 [0.92, 0.97]	0.03 [0.00, 0.35]
Main effect of Strategy						
F (1, 18)	0.03	0.14	1.49	0.74	0.27	0.78
*p*	0.876	0.715	0.238	0.401	0.611	0.388
η_p_^2^ [95% CI]	0.00 [0.00, 0.01]	0.01 [0.00, 0.09]	0.08 [0.00, 0.45]	0.04 [0.00, 0.30]	0.02 [0.00, 0.17]	0.04 [0.00, 0.30]
Interaction effect						
F (1, 18)	0.78	0.00	0.04	0.75	0.69	0.20
*p*	0.390	0.963	0.846	0.398	0.417	0.659
η_p_^2^ [95% CI]	0.04 [0.00, 0.24]	0.00 [0.00, 0.00]	0.00 [0.00, 0.02]	0.04 [0.00, 0.21]	0.04 [0.00, 0.23]	0.01 [0.00, 0.08]

*Note:* Bold *p* values indicate *p* < 0.05.

Abbreviations: η_p_^2^, partial eta squared; CE A, confidence ellipse area; HBS, hold‐based aiming strategy group; MV, mean velocity; R, resultant; SD, standard deviation; TBS, timing‐based aiming strategy group; X, cross‐shooting direction; Y, shooting direction.

**TABLE 5:** Descriptive statistics of postural control variables for the rear leg (mean ± SD) in both the hold and timing groups during the holding and shooting tasks, and the two‐way mixed ANOVA (Strategy, Task and Strategy × Task) and post hoc pairwise comparisons summary statistics.

	95% CE A [mm^2^]	R MV [mm/s]	X SD [mm]	X MV [mm/s]	Y SD [mm]	Y MV [mm/s]
All (*n* = 20)						
Holding	5.90 ± 2.20	9.26 ± 2.97	0.38 ± 0.21	2.44 ± 1.07	2.26 ± 0.65	8.35 ± 2.84
Shooting	1.35 ± 0.47	9.84 ± 2.32	0.15 ± 0.05	2.19 ± 0.61	0.81 ± 0.22	7.94 ± 2.10
HBS (*n* = 11)						
Holding	6.77 ± 2.03	10.04 ± 3.30	0.40 ± 0.21	2.73 ± 1.21	2.40 ± 0.64	9.05 ± 3.14
Shooting	1.38 ± 0.47	10.24 ± 2.24	0.16 ± 0.04	2.38 ± 0.56	0.78 ± 0.20	8.19 ± 2.07
TBS (*n* = 9)						
Holding	4.82 ± 2.00	8.30 ± 2.34	0.35 ± 0.22	2.08 ± 0.80	2.10 ± 0.67	7.49 ± 2.29
Shooting	1.31 ± 0.49	9.35 ± 2.44	0.14 ± 0.06	1.96 ± 0.63	0.85 ± 0.24	7.64 ± 2.22
Main effect of Task						
F (1, 18)	126.50	2.05	93.26	3.14	155.58	0.72
*p*	**<** **0.001**	0.169	**<** **0.001**	0.093	**<** **0.001**	0.408
η_p_^2^ [95% CI]	0.88 [0.83, 0.92]	0.10 [0.00, 0.62]	0.84 [0.77, 0.90]	0.15 [0.00, 0.39]	0.90 [0.85, 0.94]	0.04 [0.00, 0.19]
Main effect of Strategy						
F (1, 18)	3.69	1.43	0.53	4.04	0.39	1.02
*p*	0.071	0.248	0.477	0.060	0.540	0.325
η_p_^2^ [95% CI]	0.17 [0.01, 0.42]	0.07 [0.00, 0.37]	0.03 [0.00, 0.25]	0.18 [0.01, 0.53]	0.02 [0.00, 0.19]	0.05 [0.00, 0.30]
Interaction effect						
F (1, 18)	5.67	0.96	0.00	0.33	2.55	1.45
*p*	**0.029**	0.340	0.977	0.571	0.128	0.244
η_p_^2^ [95% CI]	0.24 [0.03, 0.53]	0.05 [0.00, 0.22]	0.00 [0.00, 0.00]	0.02 [0.00, 0.16]	0.12 [0.00, 0.38]	0.08 [0.00, 0.25]

*Note:* Bold *p* values indicate *p* < 0.05.

Abbreviations: η_p_^2^, partial eta squared; CE A, confidence ellipse area; HBS, hold‐based aiming strategy group; MV, mean velocity; R, resultant; SD, standard deviation; TBS, timing‐based aiming strategy group; X, cross‐shooting direction; Y, shooting direction.

A Strategy × Task interaction effect was noted in rear leg COP 95% CE A. Pairwise comparisons revealed that 95% CE A was higher in Holding compared to Shooting in both groups, yet the magnitude of this difference was larger in HBS (HBS: *p* < 0.001 and *d* = 7.18 [6.52, 9.16]; TBS: *p* < 0.001 and *d* = 4.23 [3.97, 5.18]). Whole body and front leg postural control magnitudes did not show interaction effects (Tables 3, 4, 5).

**FIGURE 2:**
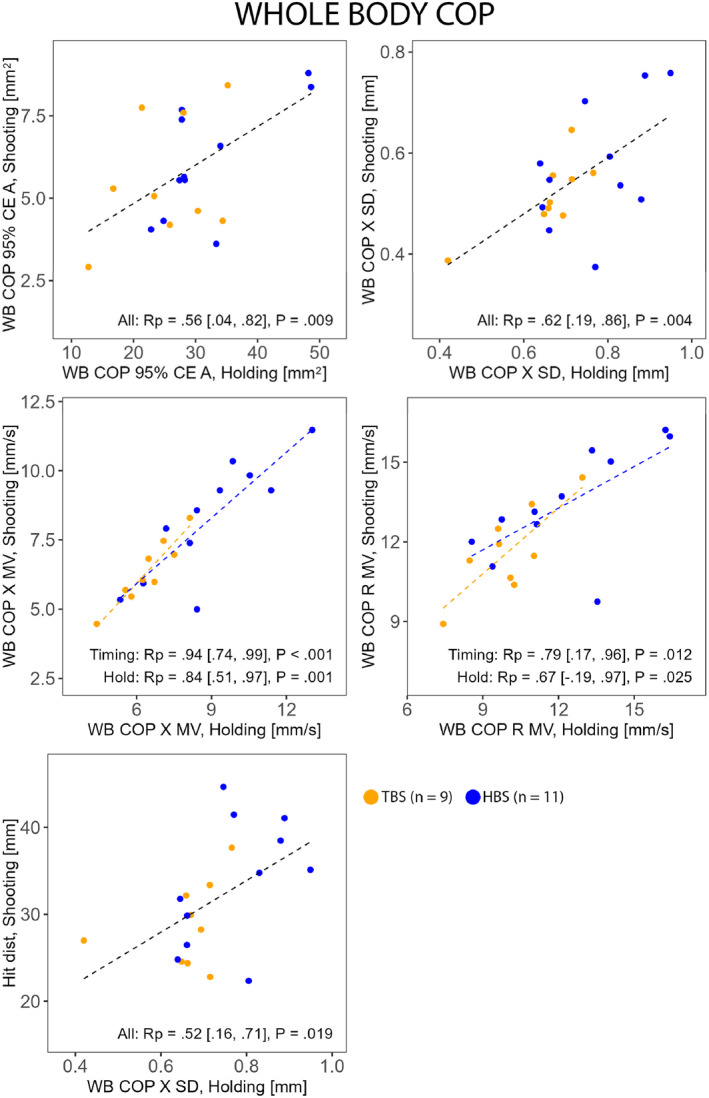
Correlations (Rp Pearson and Rs Spearman) between selected corresponding whole body (WB) postural control measures during Holding and Shooting and between whole body postural control measures during Holding and shooting performance (Hit dist) in HBS (hold‐based aiming strategy group) and TBS (timing‐based aiming strategy group). CE A, confidence ellipse area; MV, mean velocity; R, resultant; SD, standard deviation; X, horizontal (left–right).

**FIGURE 3:**
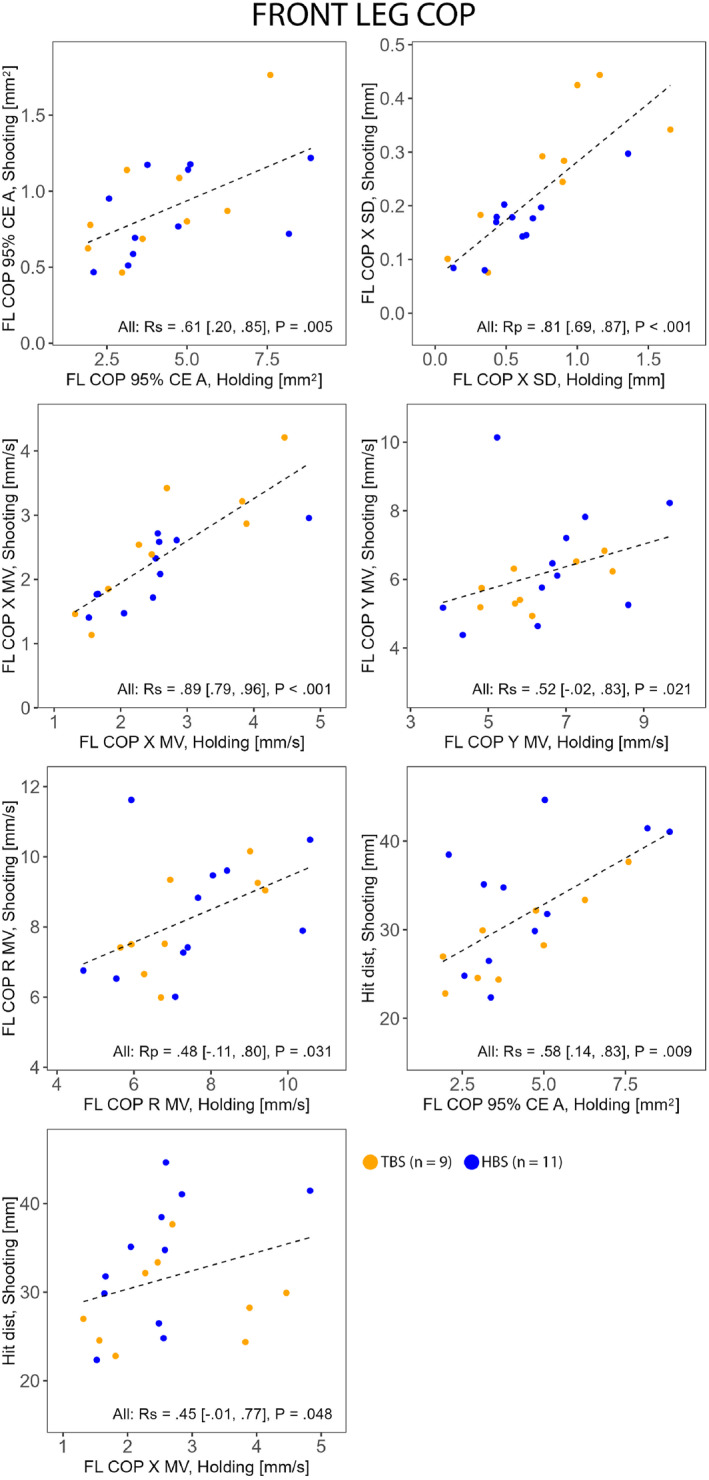
Correlations (Rp Pearson and Rs Spearman) between selected corresponding front leg (FL) postural control measures during Holding and Shooting and between front leg postural control measures during Holding and shooting performance (Hit dist) in HBS (hold‐based aiming strategy group) and TBS (timing‐based aiming strategy group). CE A, confidence ellipse area; MV, mean velocity; R, resultant; SD, standard deviation; X, horizontal (left–right); Y, vertical (up–down).

**FIGURE 4:**
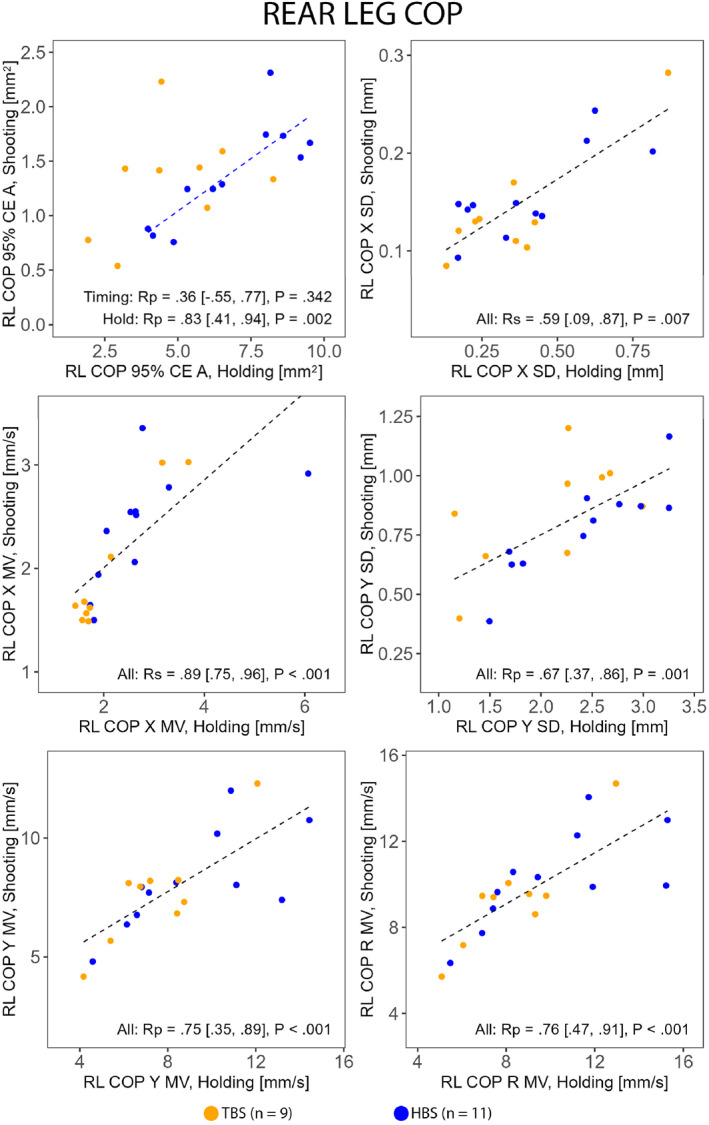
Correlations (Rp Pearson and Rs Spearman) between selected corresponding rear leg (RL) postural control measures during Holding and Shooting in HBS (hold‐based aiming strategy group) and TBS (timing‐based aiming strategy group). CE A, confidence ellipse area; MV, mean velocity; R, resultant, SD, standard deviation, X, horizontal (left–right); Y, vertical (up–down).

Whole body (Figure 2) and front leg (Figure 3) postural control magnitudes in Holding were associated with their counterparts in Shooting and with shooting performance. The complementary analysis between shooting performance and front leg COP 95% CE A with HBS and TBS separated revealed a very strong association in TBS (Rp = 0.87 [0.40, 0.98] and *P* = 0.003) but not in HBS (Rp = 0.55 [−0.18, 0.82] and *P* = 0.078). Rear leg postural control magnitudes in Holding were associated with their counterparts in Shooting (Figure 4) but not with shooting performance. Notably, only HBS exhibited associations between the tasks in rear leg COP 95% CE A (Figure 4). For all correlations, see Supporting Information S1: Supplemental Material.

## 4 Discussion

This study investigated biathlon standing shooting performance by focusing on the stability of hold and postural control during static holding (Holding) and biathlon standing shooting (Shooting) tasks. The aim was to understand the associations between these tasks and the impact of aiming strategy.

Aiming strategy influenced stability of hold but had mixed effects on postural control. Contrary to the hypothesis, TBS generally exhibited lower or similar aiming point movement. However, the magnitude of the differences between Holding and Shooting, as well as the differences within each task, varied between the groups depending on the specific measure. Although most postural control magnitude measures did not differ between groups, TBS exhibited lower resultant and cross‐shooting direction mean velocities of the whole body COP contrary to the hypothesis. HBS showed a greater difference between the tasks in the rear leg COP confidence ellipse area. Although previous research has indicated that during standing shooting, biathletes using a hold‐based strategy have better stability of hold and postural control magnitudes do not differ depending on strategy (Köykkä et al. 2021), the current findings suggest that these effects may be more complex than hypothesised.

As hypothesised, shooting performance was linked to better stability of hold and lower postural control magnitudes during Holding. However, the link was clearer in TBS, who showed a very strong association between front leg postural control magnitude and shooting performance. Stability of hold measures across tasks were correlated only in HBS, suggesting similar aiming dynamics. The absence of correlations in TBS suggests that they may require a more task‐specific test. Postural control magnitudes showed moderate to strong correlations between tasks, indicating that postural control magnitudes during Holding can reflect those also in Shooting. Although these findings support the study's hypotheses, the links between static holding and shooting have not been directly investigated in previous studies. The observed differences between strategy groups in the hold test measures associated with shooting performance, as well as in task‐to‐task correlations, highlight the need for further research to deepen the understanding upon the current findings.

### 4.1 Effects of Strategy

This study reinforces the evidence that biathletes using different aiming strategies exhibit differences in their stability of hold measures. However, not all stability of hold measures were affected by aiming strategy in this study and some discrepancies exist compared to Köykkä et al. (2021). In that study, biathletes using the timing‐based aiming strategy generally displayed greater aiming point movement than those using the hold‐based strategy. In contrast, the present results showed the opposite or no differences. A possible explanation for this discrepancy could be variation in approach velocities between the studies: HBS in the present study demonstrated similar mean approach velocity as the hold‐based aiming strategy group in the study by Köykkä et al. (2021) (57 mm/s vs. 55 mm/s) but approach velocity in TBS was lower compared to the timing‐based aiming strategy group in that study (111 vs. 144 mm/s). It is noteworthy that current research categorises aiming strategies into two groups, but these strategies may exist on a continuum, though that is beyond the scope of this study. Another possible explanation could be the differing shooting performance levels of the athletes in each group. In Köykkä et al. (2021), those using the timing‐based and hold‐based strategies hit the target on average 38 and 33 mm from the centre, respectively. In this study, the hit point distances were 31 mm (TBS) and 34 mm (HBS). Therefore, as TBS showed better shooting performance, that might partly explain the differences in stability of hold measures between the studies.

Velocity metrics representing whole body postural control magnitudes, not examined in Köykkä et al. (2021), were influenced by aiming strategy. TBS generally exhibited lower whole body postural control magnitudes in the cross‐shooting direction during both tasks. Moreover, rear leg postural control magnitude—specifically the confidence ellipse area, also not covered in Köykkä et al. (2021)—was higher during Holding in HBS compared to TBS. These findings indicate that HBS had to correct for whole body cross‐shooting line sway with their ankles to a greater extent just before triggering compared to TBS. It is possible that TBS athletes achieve their desired stability earlier during their approach to the target rather than adjusting in the final 0.6 s leading up to triggering. The higher rear leg postural control magnitude during Holding in HBS suggests that they used the rear leg ankle more heavily to control postural sway in Holding than TBS. However, during Shooting, both groups used the rear leg to a similar extent to manage sway. Whether these are adaptive differences caused by the respective aiming strategy remains speculative with the results of the present study, and thus represents an area for future research.

Aiming strategy did not significantly impact front leg postural control magnitudes in either task, which is consistent with Köykkä et al. (2021). This could be explained by the front leg's role in supporting the front arm and, consequently, the rifle on the hip. This likely limits the front leg's involvement in controlling sway. Therefore, any postural control efforts made through the front leg might cause undesired rifle movements, irrespective of the aiming strategy.

### 4.2 Associations of Shooting Performance With Stability of Hold and Postural Control

Previous studies using a static hold test in biathletes have not linked their results to shooting performance (Sadowska et al. 2019; Sadowska et al. 2020). In this study, stability of hold during Holding, particularly confidence ellipse area, was associated with shooting performance irrespective of aiming strategy, indicating that a smaller hold area was linked to more accurate shooting. Thus, static stability of hold in the standing posture appears to be a prerequisite for biathlon standing shooting success.

Despite finding significant correlations between shooting performance and whole body and front leg cross‐shooting line postural control magnitudes during Holding, these results should be treated with caution due to value dispersion (Figures 2 and 3). However, the complementary analysis showed a very strong link between front leg postural control magnitude and shooting performance in TBS, indicating that lower front leg postural control magnitude may have also contributed to better shooting for them. Thus, some strategy‐related differences regarding how a good shooting performance is achieved might exist.

### 4.3 Associations Between the Tasks in the Corresponding Stability of Hold and Postural Control Magnitude Measures

Although previous studies employing a static hold test in biathletes (Sadowska et al. 2019; Sadowska et al. 2020) have not investigated connections to shooting technique, the present study provides novel information. The results showed that stability of hold during Holding is strongly associated with stability of hold during Shooting only in HBS. This may indicate that aiming dynamics during holding align closely with the practices of HBS during their shooting. In contrast, static holding is likely too different from TBS athletes' aiming dynamics during their shooting. Consequently, TBS might benefit from a more strategy‐specific test to assess their ability to control aiming point movements during shooting in isolation, such as simulating their dynamic approach but excluding the trigger pull. Both groups showed significant associations in the vertical mean velocity of the aiming point between the tasks. However, caution is warranted in interpreting these results due to value dispersion and the correlation coefficient's confidence interval bounds extending on both sides of zero, especially in the timing group (Figure 1).

In the present study, moderate to very strong associations between the tasks were observed in various postural control magnitude measures. This generally implies that, regardless of aiming strategy, postural control measured during Holding would provide a good indication of that in Shooting. However, it is noteworthy that, for whole body postural control, associations between the tasks were observed exclusively in the cross‐shooting line magnitudes. This could be partly explained by the previously described interpretation of the whole body postural control magnitude variables (see METHODS—Variables). For example, as noted by Winter et al. (1996), the rear hip abductor activity raises the pelvis on the opposite side, thereby increasing the percentage of weight on the rear limb. In the context of biathlon standing shooting posture, this elevation influences the natural height of the aiming point due to the connection between the pelvis, the front arm and the rifle.

Speculatively, due to the nature of Holding, the natural aiming height is likely established before the start of the analysed 5‐s window, allowing for only continuous microadjustments. Conversely, during Shooting, the brief 0.6‐s window does not permit many of these up‐and‐down adjustment cycles. This mechanical difference between the tasks supports the hypothesis that whole body COP oscillations in the shooting line may relate to aiming point height adjustments and could explain why these measures are not correlated across tasks. Future research could investigate how aiming point height is controlled in the standing shooting posture and whether it relates to whole body COP movements along the shooting line during shooting.

Furthermore, a distinct pattern was observed between the groups in the rear leg confidence ellipse area, with a strong association between the tasks in HBS and an absence of association in TBS. This suggests that HBS may have a stronger link across tasks in rear leg postural control magnitudes than TBS and further highlights differences in postural control associated with aiming strategies.

### 4.4 Limitations

A small sample size is a common challenge in sports science due to the limited number of high‐performance athletes available (Skorski and Hecksteden 2021). This study faced similar limitations but managed to include 81% of all eligible biathletes in the country. Recommended efforts were made to minimise uncertainties, such as averaging multiple trials for each participant, using standardised test protocols with athletes familiarised to them, and showing confidence intervals for bootstrapped effect sizes (Hecksteden et al. 2022; Skorski and Hecksteden 2021).

This study involved tier 3 and 4 level athletes, which may limit the generalisability of the findings to world class (tier 5) athletes. Although recruiting them is challenging, it is acknowledged that performance level could influence the results. Notably, there is a lack of research on aiming strategies in biathlon shooting among tier 5 athletes.

Additionally, conducting shooting as dry firing and having participants wear running sneakers in a laboratory environment may limit the generalisability of the findings to biathlon shooting in natural biathlon training and competition environments. It is important to note that postural control and aiming point movements were evaluated before triggering, which excludes the recoil response. However, the use of running sneakers could potentially affect postural control magnitudes compared to using ski boots and standing on roller skis or skis. Although this may impact the correlation coefficients between postural control magnitudes and shooting performance, it is unlikely to influence the comparisons between aiming strategies and task‐to‐task correlations as all participants wore running sneakers in both tasks.

## 5 Conclusion

Static stability of hold is an essential skill for biathletes. However, this research highlights the critical role of aiming strategies in biathlon standing shooting and underscores the importance of considering these strategies in academic studies and coaching practices. For biathletes using the hold‐based aiming strategy (HBS), aiming dynamics during a static holding task closely mirror those during their shooting, making static testing a reliable indicator of their stability of hold during shooting. In contrast, although static stability of hold remains important for biathletes using the timing‐based aiming strategy (TBS), their dynamic aiming approach necessitates tailored evaluations that incorporate dynamic movement and timing patterns specific to their approach. Furthermore, postural control magnitudes in the cross‐shooting direction during the static holding task generally are associated with those seen during shooting. Thus, postural control measured during a static holding task can provide a good indication of that in biathlon standing shooting as well. However, differences in whole body postural control mechanics, particularly in metrics related to the shooting direction, should be acknowledged.

As the first study to establish a link between a static holding test and biathlon standing shooting performance, the static holding test protocol provides an efficient way to assess key characteristics such as isolated stability of hold and postural control. It can help coaches and biathletes track training progress, adjust training strategies, and fine‐tune rifle settings or shooting posture to optimise these critical factors. By targeting stability of hold and postural control, focused interventions can lead to improvements in biathlon shooting performance. Future research could explore the impact of static hold training, as well as the effects and time frames of optimising rifle settings and shooting posture, on biathlon standing shooting outcomes.

## Ethics Statement

The study was approved by the University of Jyväskylä Ethics Committee (6 June 2019).

## Conflicts of Interest

The authors declare no conflicts of interest.

## Supporting information


Supporting Information S1

## Data Availability

Original data are available from the corresponding author upon a reasonable request.

## Appendix 1 Details of Centre of Pressure Coordinates Computation From Force Platform Data

1. The centre of pressure (COP) coordinates were computed individually for the front and rear legs by deriving the COP displacement from the force plate origin using the formula:
  $$d=\frac{M}{F}$$
  Here, d represents COP displacement, M the three‐dimensional moment ($M_{x}$; $M_{y}$ and $M_{z}$) and F the vertical ground reaction force (vGRF).
2. The whole body COP coordinates were computed utilising the moment signals respective to the defined origin of the dual force platform setup and vGRFs of each force platform using the formula:
  $$d_{\text{tot}}=\frac{F_{1}\times \left(d_{1}+d_{fp1}\right)+F_{2}\times \left(d_{2}+d_{fp2}\right)}{F_{1}+F_{2}}$$
  Here, $d_{\text{tot}}$ represents the COP displacement from the defined origin of the dual force platform system, $F_{1}$ and $F_{2}$ are the vGRFs of each force platform, $d_{1}$ and $d_{2}$ are the calculated COP displacements of each force platform and $d_{fp1}$ and $d_{fp2}$ are the distances of each force platform origins from the defined origin of the dual force platform system.

## Appendix 2 Details of Aiming Point Trajectory Computation From Marker Data

1. A global coordinate system is defined with the origin positioned at a point 50 m away from the targets. The *X*‐axis is oriented perpendicular to the shooting direction (left–right), the *Y*‐axis runs parallel to the shooting direction (forwards–backwards) and the *Z*‐axis is perpendicular to the plane formed by the *X* and *Y* axes (up–down).
2. In this setup, the targets lie in a plane perpendicular to the shooting direction, maintaining a constant *Y*‐coordinate of 50,000 mm. Consequently, the intersection of the aiming point with this plane can be identified as point $P_{3}=\left(x,50000,z\right)$.
3. The two markers attached to the rifle barrel, $P_{1}=\left(P_{1x},P_{1y},P_{1z}\right)$ and $P_{2}=\left(P_{2x},P_{2y},P_{2z}\right)$, form the barrel line, corresponding to the aiming direction.
4. A vector parallel to the barrel line, $P_{12}$, is formed using points $P_{1}$ and $P_{2}$:
  $$P_{12}=P_{2}-P_{1}$$
5. A vector from the barrel to the target plane, $P_{23}$, is formed using points $P_{2}$ and $P_{3}$:
  $$P_{23}=P_{3}-P_{2}$$
6. At the aiming point, $P_{23}={tP}_{12}$, meaning vectors $P_{23}$ and $P_{12}$ are parallel, and the former is obtained by multiplying the latter by a scalar t.
7. The coordinates of points $P_{1}$, $P_{2}$ and $P_{3}$ are substituted into the equation, resulting in:
  $$\left(x-P_{2x}\right)i+\left(50000-P_{2y}\right)j+\left(z-P_{2z}\right)k=t\left(P_{2x}-P_{1x}\right)i+t\left(P_{2y}-P_{1y}\right)j+t\left(P_{2z}-P_{1z}\right)k$$
8. Setting i, j and k components equal, a system of three equations with three unknowns is obtained:
  $$\left\{\begin{array}{c} x-P_{2x}=t\left(P_{2x}-P_{1x}\right)\\ 50000-P_{2y}=t\left(P_{2y}-P_{1y}\right)\\ z-P_{2z}=t\left(P_{2z}-P_{1z}\right) \end{array}\right.$$
9. Solving this system yields the values of t, x and z, where x represents the horizontal and z the vertical coordinates of the aiming point.
10. The targets lie in a plane perpendicular to the shooting direction, maintaining a constant *Y*‐coordinate of 50,000 mm. Consequently, the intersection of the aiming point with this plane can be identified as point $P_{3}=\left(x,50000,z\right)$.
11. The two markers attached to the rifle barrel, $P_{1}=\left(P_{1x},P_{1y},P_{1z}\right)$ and $P_{2}=\left(P_{2x},P_{2y},P_{2z}\right)$, form the barrel line, corresponding to the aiming direction.
12. A vector parallel to the barrel line, $P_{12}$, is formed using points $P_{1}$ and $P_{2}$:
  $$P_{12}=P_{2}-P_{1}$$
13. A vector from the barrel to the target plane, $P_{23}$, is formed using points $P_{2}$ and $P_{3}$:
  $$P_{23}=P_{3}-P_{2}$$
14. At the aiming point, $P_{23}={tP}_{12}$, meaning vectors $P_{23}$ and $P_{12}$ are parallel, and the former is obtained by multiplying the latter by a scalar t.
15. The coordinates of points $P_{1}$, $P_{2}$ and $P_{3}$ are substituted into the equation, resulting in:
  $$\left(x-P_{2x}\right)i+\left(50000-P_{2y}\right)j+\left(z-P_{2z}\right)k=t\left(P_{2x}-P_{1x}\right)i+t\left(P_{2y}-P_{1y}\right)j+t\left(P_{2z}-P_{1z}\right)k$$
16. Setting i, j and k components equal, a system of three equations with three unknowns is obtained:
  $$\left\{\begin{array}{c} x-P_{2x}=t\left(P_{2x}-P_{1x}\right)\\ 50000-P_{2y}=t\left(P_{2y}-P_{1y}\right)\\ z-P_{2z}=t\left(P_{2z}-P_{1z}\right) \end{array}\right.$$
17. Solving this system yields the values of t, x and z, where x represents the horizontal and z the vertical coordinates of the aiming point.
